# GeoGraphNetworks: A comprehensive benchmark dataset for accurate and scalable graphical representations

**DOI:** 10.1016/j.dib.2026.112655

**Published:** 2026-03-06

**Authors:** Harish Sharma, Peter Mooney, Edgar Galván

**Affiliations:** Naturally Inspired Computation Research Group and Hamilton Institute, Department of Computer Science, National University of Ireland Maynooth, Maynooth, Ireland

**Keywords:** Geographic information system (GIS), Transportation and river networks, Graph representation of infrastructure, Open Geospatial Data, NetworkX Graphs

## Abstract

Accurate graphical representations of real-world systems are essential for research in fields such as transportation, urban planning, ecology, and network science. While ESRI Shapefiles are a widely used source of geospatial vector data, converting them into topologically correct and usable network format requires significant technical expertise and computational resources. To address this challenge, we present the GeoGraphNetworks data repository, which contains 110 validated spatial networks and offers comprehensive and publicly accessible spatial network resources for the road and rail networks of the United States of America and the road and river networks of Great Britain. This data repository eliminates the need for users to perform complex geospatial processing by providing workable graph representations in JSON and XLSX formats. Each network includes detailed geographic information such as node coordinates (latitude and longitude), edge connectivity, and edge lengths in kilometres. By reducing preprocessing overhead and enabling immediate application across multilingual programming environments, GeoGraphNetworks lowers technical barriers and supports reproducible, scalable spatial network research across disciplines.

Specifications Table

**Table:** 

Subject	Computer Sciences
Specific subject area	Graph networks dataset derived from validated ESRI shapefiles for transportation urban planning and network science research
Type of data	Graph data (JSON objects); Spreadsheet tables (XLSX edge lists); Python Scripts; Jupyter Notebooks
Data collection	Derived from official geospatial datasets (ESRI Shapefiles) of road, rail, and river networks. Shapefiles were obtained from national mapping agencies, Ordnance Survey (Open Roads and Open Rivers) for Great Britain; U.S. Census Bureau (TIGER/Line and North American Rail Network) for USA and processed using Python libraries (GeoPandas, Shapely) to extract graph edges.
Data source location	Road and rail networks across all 50 U.S. states (plus D.C. and territories); road and river networks across Great Britain. Data compiled at Maynooth University, Ireland (National University of Ireland, Maynooth).
Data accessibility	Repository name: GeoGraphNetworks Data identification number: https://doi.org/10.6084/m9.figshare.27284859 Direct URL to data: https://figshare.com/articles/dataset/GeoGraphNetworks_Great_Britain_s_Web_of_Roads_Rivers/27284859
Related research article	None

## 1 Value of the Data

**Fig. 1:**
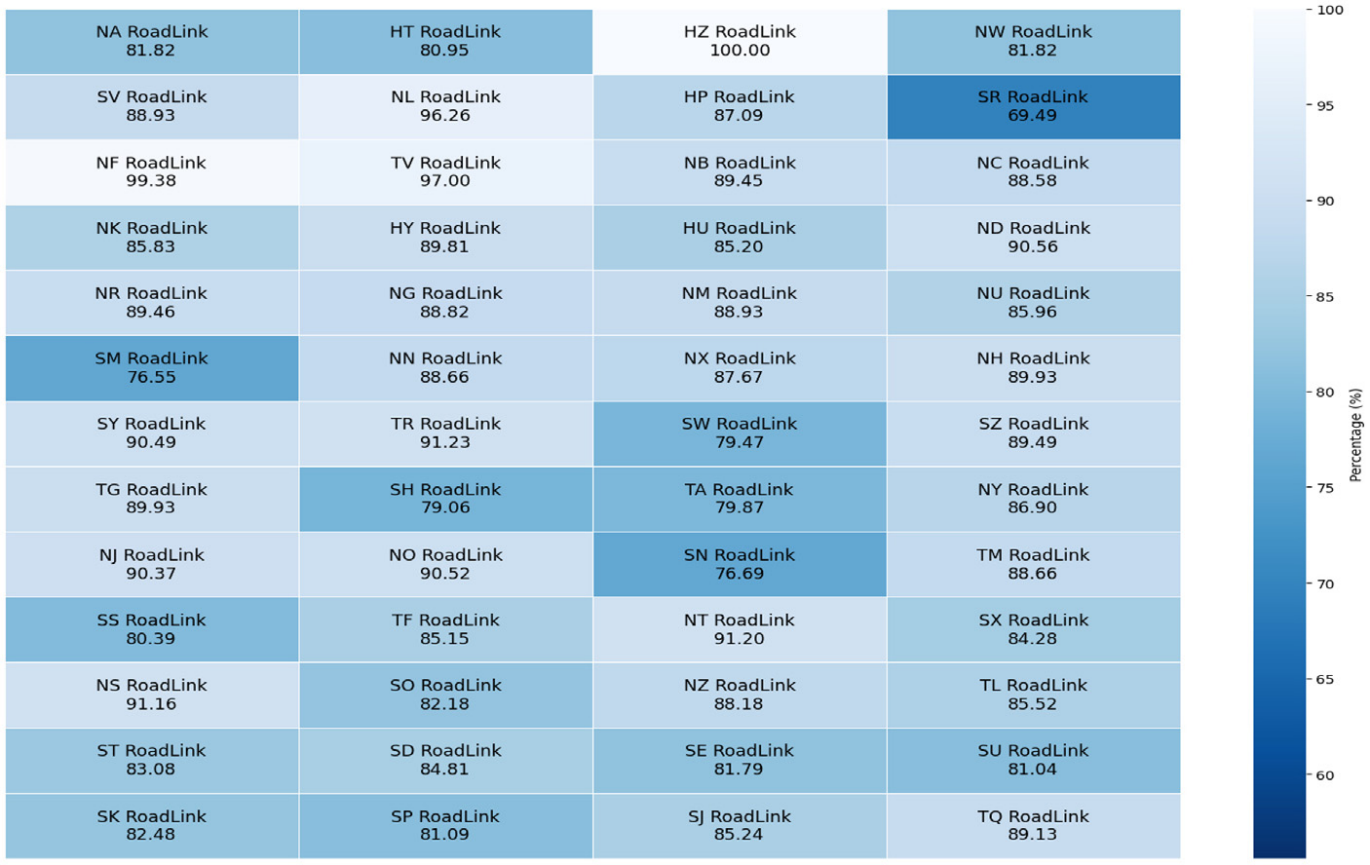
Proportion of nodes in each generated graph that match the junction points in ESRI Shapefiles for the 52 road networks from GB in this study.
• **Cross-Platform Reusability:** Networks are distributed in standardized, widely supported formats (e.g., JSON and XLSX edge lists), allowing them to be readily used across different programming environments and tools, including NetworkX, OSMnx, and common GIS software. This flexibility enables straightforward integration into diverse analytical workflows and makes the datasets accessible to users with varying levels of technical expertise.
• **Broad Application Scope:** The GeoGraphNetworks [1] data can be applied to a wide range of scientific and engineering problems. Example reproducible workflows include: (i) shortest-path and routing benchmarks using edge weights for travel distance/time, (ii) graph analytics such as centrality and resilience analysis using network topology and connectivity fields, and (iii) optimization and learning tasks including vehicle routing, multi-criteria path planning, and graph machine learning model training. The dataset has already been used in real-world routing and optimization studies, including K-Most Diverse Near-Shortest Paths [3] and multi-criteria vehicle routing on complex road networks [4], demonstrating its practical utility for algorithm development and benchmarking.
• **Supports Reproducible Research:** Each network includes explicit node and edge attributes (e.g., coordinates and segment distances) and is distributed in standardized formats together with open-source Python scripts and Jupyter Notebooks (GitHub) that implement the full preprocessing workflow. These resources enable users to regenerate the networks directly from the original shapefiles and reproduce all transformation steps without ambiguity.
• **Expandable and Community-Driven:** The dataset is maintained under a Creative Commons license and open to contributions, with plans for regular updates and user-suggested additions through GitHub or email.

• **Pre-processed, Standardized, and Scalable Format:** As noted in the abstract, GeoGraphNetworks [1] provides 110 analysis-ready undirected weighted spatial networks in standardized node and edge formats, enabling immediate use without geospatial preprocessing. The consistent schema across all regions allows networks to be easily combined programmatically to construct larger, connected systems, for example, multiple state-level road networks can be merged to form regional or national-scale graphs, supporting scalable analyses from local to country-wide levels.
• **Derived from authoritative national mapping agencies:** All networks originate from official government sources, including Ordnance Survey for Great Britain (GB) and United States Census Bureau, ensuring national coverage, consistent formatting, and high data reliability. For GB, the availability of junction point ESRI Shapefiles enables quantitative validation: we measured the proportion of generated graph nodes that align with official junction locations. As shown in Fig. 1, alignment rates are consistently high across the 52 road networks (e.g., >89 % for the largest network with 373,079 nodes and 445,192 edges, and 80.96 % for the GB river network), demonstrating accurate node extraction and reliable intersection representation. Additional validation includes visual cross-validation [2], basic graph analyses, and successful reconversion of graph components back to ESRI Shapefile format, supporting structural consistency and reproducibility.

## 2 Background

Geographical systems play a vital role in providing accurate and comprehensive data for analysis, mapping, and decision-making in various fields such as emergency response, urban planning, transportation, and environmental management [3,4]. A fundamental prerequisite for geographical systems is the availability of precise data that accurately represents geographic objects and features, including their positions, areas, and distances from other objects. For a long time, one of the most dependable data sources for collecting and storing such geographic objects and features has been ESRI Shapefiles. These Shapefiles store geospatial data, such as buildings, traffic signals, railway lines, roads, rivers, and more, using a vector data format that utilizes different geometries like polygons, polylines, and points for representation [5].

Python packages NetworkX [6] and OSMnx [7] are probably the most popular approaches in industry for creating and analysing real world graphical networks. However, generating such networks from raw geospatial data remains non-trivial, as these libraries require data to be converted into explicit node and edge topologies. While OSMnx supports Shapefile based inputs, additional preprocessing is often required to ensure correct network connectivity. Our earlier study [5] introduced an open-source transformation pipeline for constructing such networks, it required GIS expertise, Python proficiency, and substantial computational resources. The networks provided here remove these barriers by offering ready-to-use graph representations.

## 3 Data Description

For GB, a total of 52 road networks are provided. The OS Open Roads data do not include Shapefiles for tiles HW and HX because no road geometries exist in those areas (see Fig. 3). These tiles are therefore intentionally absent from the dataset and are explicitly recorded as missing in the dataset manifest to enable programmatic detection. In contrast, the OS Open Rivers dataset provides a single ESRI Shapefile covering the entire river network of GB. For the USA, primary and secondary road networks are provided for all 50 states, Washington, D.C., and five territories, yielding 56 road networks. Additionally, a single national rail network connects the continental United States with parts of Canada.

In total, the dataset comprises 220 files: 110 in Excel (.xlsx) format and 110 in JSON format, representing 52 GB road networks and one GB river network, as well as 56 USA road networks and one USA rail network. These files constitute the processed graph representations distributed with this dataset. The original source ESRI Shapefiles are not included as primary data products, but are provided separately on the project GitHub repository for reference and visualization purposes. The JSON files contain graph objects generated using the Python library NetworkX [6] and are ready for immediate use without additional preprocessing. The networks are stored as undirected weighted graphs unless otherwise specified.

The XLSX files contain data in the format shown in Table 1 and provided to enable users to create graph networks using various tools and programming languages, ensuring flexibility across platforms. Nodes in each graph are assigned unique IDs and are positioned according to their original geospatial locations. For instance, (539,154.5, 200,022.19) from Table 1 represents a single node with the original position as per British National Grid format (EPSG: 27,700, meters). Table 2 provides a detailed data dictionary describing all columns, units, and coordinate reference systems used for both GB and USA networks.

**Table 1:** Sample format for an edge list dataset, including the X and Y coordinates of the start and end nodes that define each edge, as well as the edge length in Kilometres. It is important to note that the coordinates in this table are provided in the British National Grid format (EPSG: 27,700, meters). A transformation to EPSG: 4326 is necessary to convert them to standard longitude and latitude coordinates.

${XCOORD}_{Start}$	$YCOORD_{Start}$	$XCOORD_{End}$	$YCOORD_{End}$	Edge Length (in Kilometres)
539,154.5	200,022.19	539,313	199,992	0.169336175
539,328.52	200,065.15	539,313	199,992	0.100025021
538,926	200,043	539,092	199,947	0.212559337
539,114.7	199,964.64	539,092	199,947	0.028748210
539,154.5	200,022.19	539,114.7	199,964.64	0.069971727
538,998.73	199,993.39	539,114.7	199,964.64	0.119807566

**Table 2:** Data Dictionary for XLSX edge-list files. Graphs are stored as undirected weighted networks, with JSON files providing ready-to-use graph objects generated using NetworkX.

**Column**	**Unit / CRS**	**Description**
$XCOORD_{Start}$	meters (EPSG:27,700 GB; EPSG:2163 USA)	Projected X/Easting coordinate of start node
$YCOORD_{Start}$	meters (EPSG:27,700 GB; EPSG:2163 USA)	Projected Y/Northing coordinate of start node
$XCOORD_{End}$	meters (EPSG:27,700 GB; EPSG:2163 USA)	Projected X/Easting coordinate of end node
$YCOORD_{End}$	meters (EPSG:27,700 GB; EPSG:2163 USA)	Projected Y/Northing coordinate of end node
Edge Length	kilometers	Physical edge distance computed from projected geometry; used as graph weight

For transparency, the visual representation of each network is provided in both ESRI Shapefile format and NetworkX [6] graph format on our GitHub page. For instance, the GB river network is illustrated both in its Shapefile view and as a simplified Graph view, highlighting the transformation from raw geospatial data to a usable network model.

**Dataset structure and naming:** Network files are named according to their geographic scope. GB road networks use Ordnance Survey two-letter tile codes (100 km × 100 km grid cells; Fig. 3), while the GB river network is provided as a single national dataset. USA networks are named using state or territory names, with a single file representing the USA rail network. This consistent naming scheme enables straightforward identification of region and network type.

**Fig. 2:**
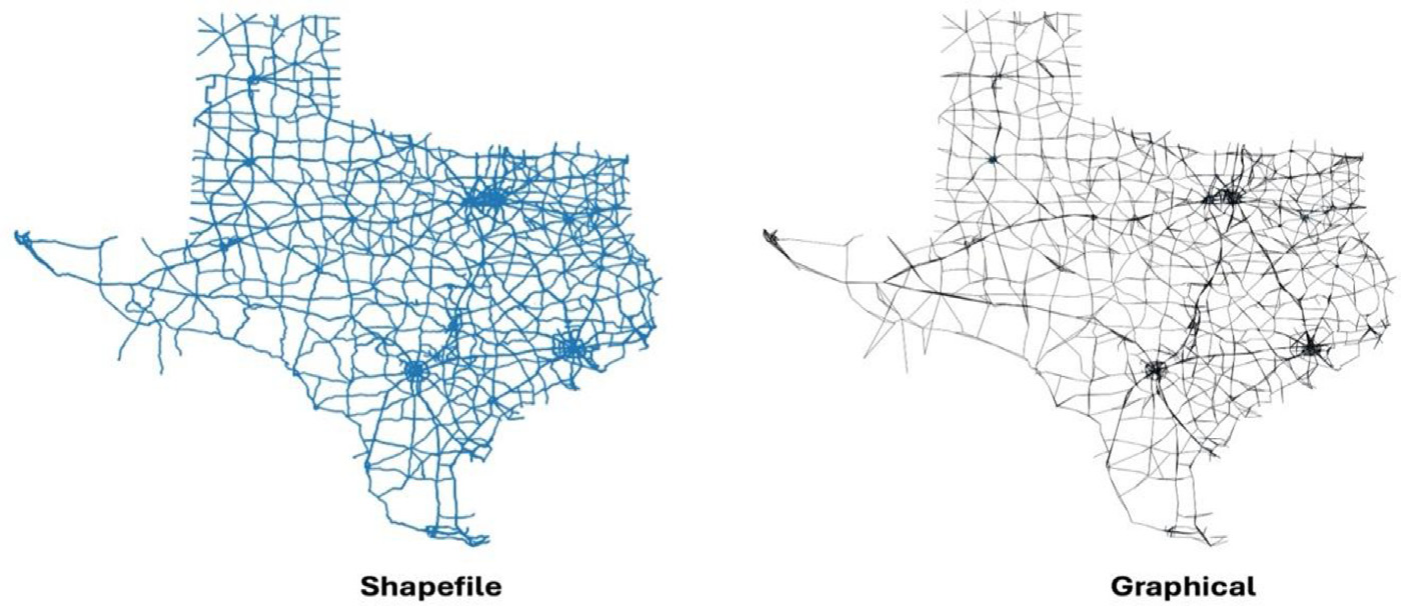
Shapefile representation of Texas (left), displaying polyline geometries. The graphical representation of Texas (right) is constructed using extracted nodes and edges in NetworkX, illustrating the spatial network structure.

**Repository and auxiliary materials:** The entire dataset is hosted under a Creative Commons (CC) license on Figshare, titled *GeoGraphNetworks*. Users can download individual network files or the whole collection as needed. In addition to the data files, a set of Jupyter Notebooks is provided on the project’s GitHub page to demonstrate how to load, visualize, and perform basic analysis on these networks. The GitHub repository also includes rendered images for each network to support quick inspection and cross-validation. In Fig. 2, a side-by-side visualization of Texas shows the cleaned and pre-processed road shapefile alongside its corresponding NetworkX graph, constructed from extracted nodes and edges, illustrating the resulting spatial network structure. This supplementary material helps users understand the data representation and ensures the datasets can be readily and correctly used in research workflows.

**Fig. 3:**
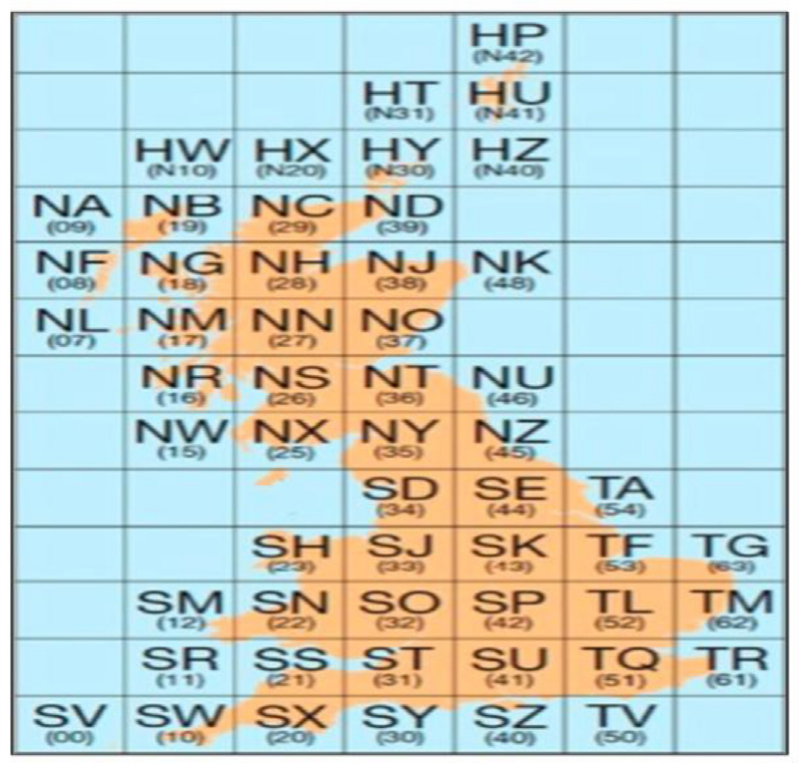
This image is sourced from the ordnance surveys official website, specifically from the OS Open Roads. It displays a map of the UK segmented into 100 km-by-100 km tiles.

## 4 Experimental Design, Materials and Methods

This section outlines the complete workflow used to produce the graphical networks introduced in the GeoGraphNetworks [1] data repository. Our goal is to illustrate the entire process, from the selection of ESRI Shapefiles for this research to the method used to transform the ESRI Shapefiles containing polyline geometries into an edge list format suitable for a graphical network. Three main phases of our workflow are as follows:

• Data gathering
• Pre-processing
• Transformation method

### 4.1 Data gathering

The initial phase of our workflow involved data gathering, a non-trivial task given the requirements such as credible data source, size, and scope of the datasets introduced. We created the following specialized factors and conditions for the data collection process in order to give a consistent and uniform procedure:

• **Selection of Country:** The United States of America (USA) and Great Britain (GB) are selected as the focus areas because their networks comprehensively cover the entire country, and open-source geospatial data is readily available. Additionally, the ease of data access and the frequent updates to repositories by the U.S Census Bureau and Ordnance Survey further establish the USA and GB as ideal choices for this study.
• **Data Source:** To ensure high quality datasets for this study, we relied solely on data repositories from reputable sources. In GB, Ordnance Survey (OS), the national mapping agency, serves the government, private sector, and public by conducting official surveys and providing accurate, up-to-date geographic data. Similarly, the US Census Bureau supports these sectors by conducting official surveys and offering precise, up-to-date geographic and demographic data, including mapping resources such as TIGER/Line files.
• **Industry-Standard Geospatial Data:** We also aimed to ensure that the ESRI Shapefiles we are using have a track record of being utilized in academic research. For instance, OS data has been cited in [8,9] and Tiger/line data has been cited in studies [10,11].
• **Availability:** All the ESRI Shapefiles used in this work are open source.
• **Coverage:** We selected ESRI Shapefiles that provide comprehensive national coverage.

To maintain manageable file sizes, OS divides the road map of GB into 100 km-by-100 km grid squares in the Open Roads dataset (see Fig. 3). This image is sourced from the Ordnance Survey official website.

### 4.2 Pre-processing

A graph is characterized as a set of edges connecting two nodes (also known as vertices), mathematically defined as a pair of sets, *G = (V, E)*, where *V* is a set of nodes and *E* is a set of Edges [12]. In a graph, each node is positioned using the longitude (position on the x-axis) and latitude (position on the y-axis) coordinates. Each edge in the graph is assigned a distance (weight), which represents the cost of moving from one node to another along that edge. The distance between two nodes $X_{\left(x_{1},y_{1}\right)}$ and $Y_{\left(x_{2},y_{2}\right)}$ is calculated using Eq. (1) where $\left(x_{1},\;y_{1}\right)$ and $\left(x_{2},y_{2}\right)$ represents the longitude and latitude coordinates of nodes X and Y, respectively.

(1) $$Distance=\;\sqrt{{\left(x_{2}-\;x_{1}\right)}^{2}+\;{\left(y_{2}-\;y_{1}\right)}^{2}}$$

As the position of the nodes and the distance along the edges depend only on the longitude and latitude coordinates, the first stage of the pre-processing phase was to remove the elevation coordinate (position on the z-axis) from the ESRI Shapefiles used in our study. Using the longitude and latitude coordinates directly in Eq. (1) will yield distances in degrees, which are typically unsuitable for most applications. To address this, conversion to practical units such as meters or kilometers, is required. To ensure that the polyline geometries stored in the ESRI Shapefiles are expressed using a meter-based projection system, which is crucial to create an appropriate graphical representation from an ESRI Shapefile, we need to extract the actual lengths (in meters) of the road segments [3,4]. The European Petroleum Survey Group (EPSG) is a non-profit organization that keeps and maintains geographic records using standard codes known as EPSG codes, which define the Coordinate Reference System (CRS) for ESRI Shapefile projections [5]. The meter-based projection codes for geographic features in the USA and GB are EPSG:2163 and EPSG:27,700, respectively. The database of EPSG codes can be searched for further details.

Data cleaning is the last stage of our pre-processing phase. The cleaning stage as it is described in [5] includes not only finding and eliminating duplicate records but also finding and eliminating multiple entries that might not make a substantial contribution to the network. The ESRI Shapefiles stores linear features like road segments, railway line segments, river watercourse using polyline geometries. These geometries are highly efficient for storing and representing real-world networks, along with all their features and attributes. However, in graphical networks, these features are expressed using a single edge between two nodes. Therefore, some features or attributes which might be very crucial to an network in ESRI Shapefile become inadequate to a graphical network. In our study, we considered a polyline inadequate when,

• One polyline lies completely within another polyline. For instance, when a new road segment is built over a pre-existing road, representing an overpass [5].
• One polyline contains a piece of another polyline (more than one consecutive point). This situation is exemplified by city road segments that are part of major highways [5].
• One polyline intersects with a same polyline at more than one location. For instance, a road segment that crosses a highway twice while running parallel to it [5].

By employing the aforementioned criteria, we eliminated a significant portion of the road segments from our graph without changing the graph's overall structure.

### 4.3 Transformation method

We describe below the complete, step-by-step process for converting a network from an ESRI Shapefile format into a cleaned, validated graphical network. All networks are generated using multiple Jupyter notebooks running simultaneously on a consistent device featuring a 12-core CPU (6 performance cores and 6 efficiency cores). While our primary implementation is in Python (code available in our GitHub repository), the core logic is language-agnostic and can be adapted to other platforms. To facilitate reproducibility, the provided implementation was developed and tested using Python 3.10 with the following libraries: GeoPandas 1.1.1, Shapely 2.1.1, Pandas 2.3.2, and NumPy 2.1.3. The preprocessing workflow is executed through a single central function that automates the complete pipeline from shapefile input to final network export, enabling consistent and repeatable results across systems. Throughout the process, any geometries that cannot be split correctly are flagged and recorded for manual inspection, ensuring the integrity of the resulting network. The major steps involved in the transformation process are as follows:

• An ESRI Shapefile containing a polyline network is a prerequisite. It can be downloaded from the U.S. Census Bureau TIGER/Line and Ordnance Survey data repositories.
• Read the respective ESRI Shapefile using GeoPandas [13].
• Using Shapely [14], drop the elevation coordinate (if present) from all polyline geometries in the network.
• Transform the EPSG code of the stored geometries to a meter-based projection system if necessary. GeoPandas [13] provides the functionality for the transformation.
• Remove all polylines that lies within another polyline. This can be achieved using the functionalities from Shapely [14].
• Extract the coordinates of the first and last node of each polyline using Shapely [14] and create a set of edges.
• Using Shapely [14], track the intersection of each polyline with others. Only if the intersecting geometry is a point, add two edges (from the first node to the intersection node, and from the intersection node to the last node) to our set of edges. This step will eliminate the inadequate polylines as per the conditions specified in our data cleaning strategy.
• Remove any duplicate edges, if formed.
• Using Shapely [14], calculate the length of each edge (in meters). All edge lengths are computed in projected meter-based CRS (EPSG:27,700 or EPSG:2163). No distances are calculated in latitude/longitude degrees.
• Download or save the network in the edge list format, using GeoPandas [13] or saving as a CSV or XLSX file for reusability.

**Fig. 4:**
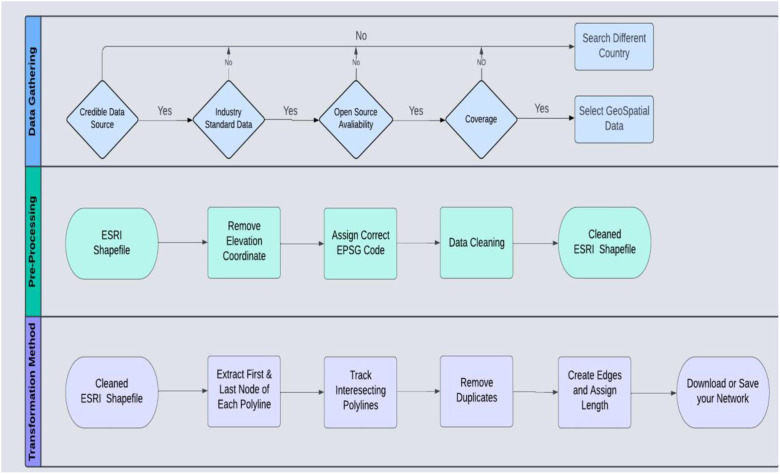
A schematic outline of the overall process.

**Fig. 5:**
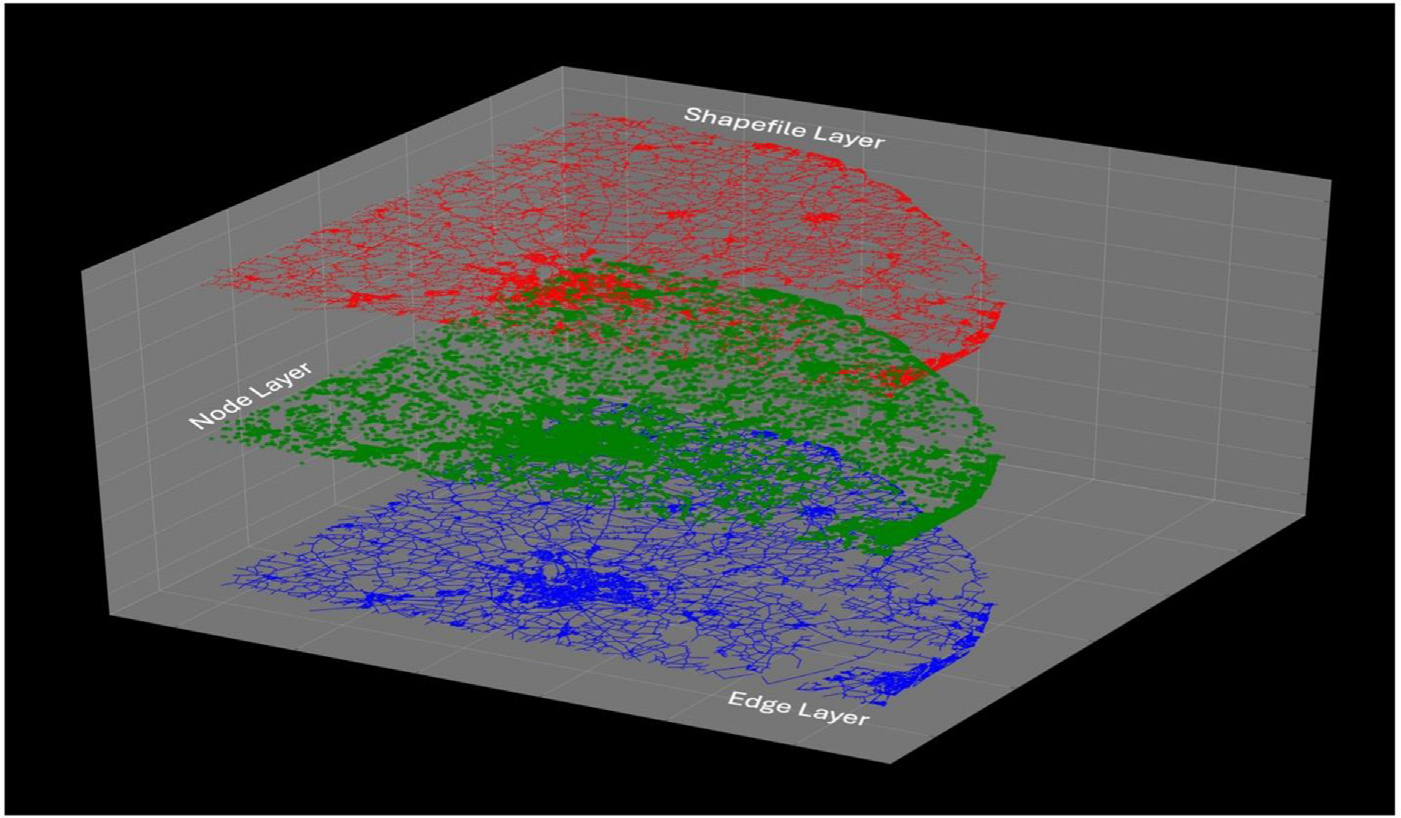
This example demonstrates the transformation method in action. The top layer represents the ESRI Shapefile, the middle layer displays the extracted nodes, and the bottom layer illustrates the edges connecting these nodes.

With data in the edge list format, graphical representations of any network can be created in multilingual programming environments. Fig. 4 provides a detailed description of the overall process and Fig. 5 is used to demonstrate a practical example of our transformation, where the first layer represents the ESRI Shapefile, the second layer shows the nodes extracted from it, and the final layer represents the edges connecting these nodes.

## Limitations

The GeoGraphNetworks [1] data repository, while comprehensive, has certain limitations stemming from its scope and source data. First, the road networks for the USA include only primary and secondary roads, more minor local roads and streets are not included, which means the dataset focuses on the main transportation backbones rather than every roadway. This choice keeps the networks to a manageable size and relevance for large-scale studies, but it may limit analyses that require fine-grained local road detail. Second, the datasets are currently limited to two countries/regions (USA and GB). Networks for other countries or regions are not part of this collection, although the framework could be extended in the future. These design choices balance dataset usability with computational tractability and are consistent with intended large-scale benchmarking use cases.

## Ethics Statement

The authors have read and follow the ethical requirements for publication in Data in Brief and confirm that the current work does not involve human subjects, animal experiments, or any data collected from social media platforms.

## CRediT authorship contribution statement

**Harish Sharma:** Writing – review & editing, Writing – original draft, Visualization, Validation, Software, Methodology, Investigation, Formal analysis, Data curation, Conceptualization. **Peter Mooney:** Writing – review & editing, Supervision, Project administration. **Edgar Galván:** Writing – review & editing, Supervision, Project administration.

## Data Availability

figshareGeoGraphNetworks: Shapefile-Derived Datasets for Accurate and Scalable Graphical Representations (Original data)
